# Measurement Invariance of Screening Measures of Anxiety, Depression, and Level of Functioning in a US Sample of Minority Older Adults Assessed in Four Languages

**DOI:** 10.3389/fpsyt.2021.579173

**Published:** 2021-02-15

**Authors:** Mario Cruz-Gonzalez, Patrick E. Shrout, Kiara Alvarez, Isaure Hostetter, Margarita Alegría

**Affiliations:** ^1^Disparities Research Unit, Department of Medicine, Massachusetts General Hospital, Boston, MA, United States; ^2^Department of Medicine, Harvard Medical School, Boston, MA, United States; ^3^Department of Psychology, New York University, New York, NY, United States; ^4^Department of Health Policy, Harvard School of Public Health, Boston, MA, United States; ^5^Department of Psychiatry, Harvard Medical School, Boston, MA, United States

**Keywords:** minority older adults, linguistic minorities, measurement invariance, anxiety, depression, level of functioning

## Abstract

Population aging in the US and its increase in racial/ethnic diversity has resulted in a growing body of literature aimed at measuring health disparities among minority older adults. Disparities in health outcomes are often evaluated using self-reported measures and, to attend to linguistic diversity, these measures are increasingly being used in languages for which they were not originally developed and validated. However, observed differences in self-reported measures cannot be used to infer disparities in theoretical attributes, such as late-life depression, unless there is evidence that individuals from different groups responded similarly to the measures—a property known as measurement invariance. Using data from the Positive Minds-Strong Bodies randomized controlled trial, which delivered evidence-based mental health and disability prevention services to a racially/ethnically diverse sample of minority older adults, we applied invariance tests to two common measures of anxiety and depression (the GAD-7 and the HSCL-25) and two measures of level of functioning (the Late-Life FDI and the WHODAS 2.0) comparing four different languages: English, Spanish, Mandarin, and Cantonese. We found that these measures were conceptualized similarly across languages. However, at the item-level symptom burden, we identified a non-negligible number of symptoms with some degree of differential item functioning. Spanish speakers reported more *worry* symptoms and less *somatic* symptoms for reasons unrelated to their psychological distress. Mandarin speakers reported more *feelings of restlessness*, and both Mandarin and Cantonese speakers reported *no interest in things* more often for reasons unrelated to their psychological distress. Mandarin and Cantonese speakers were also found to consistently report more difficulties performing physical activities for reasons unrelated to their level of functioning. In general, invariance tests have been insufficiently applied within psychological research, but they are particularly relevant as a prerequisite to accurately measure health disparities. Our results highlight the importance of conducting invariance testing, as we singled out several items that may require careful examination before considering their use to compare symptoms of psychological distress and level of functioning among ethnically and linguistically diverse older adult populations.

## Introduction

Fueled by low fertility and increased life expectancy, the population aged 65 and over is projected to increase 150% worldwide by 2050 (1). Consistent with this pattern, the US population aged 65 and over is expected to double by 2050 and to become more ethnically diverse, with racial/ethnic minority older adults projected to make up 39.1% of the 65 years and over population compared to 20.7% in 2012 (2). Since late-life mental illnesses—particularly depression—and associated comorbidities (e.g., cognitive decline and disability) are common health problems in US older adults, population aging and its increase in racial/ethnic diversity has resulted in a growing body of literature aimed at measuring health disparities in these populations (3). These studies have revealed that racial/ethnic US minority older adults are at increased risk for severity, persistence and recurrence of psychiatric disorders (4–7) and at increased risk of functional limitations, impairment and disability (8).

Notwithstanding the importance of recognizing racial/ethnic health disparities among older adults, most research studies characterizing these populations make the underlying—yet testable—assumption that the instruments measuring health outcomes are interpreted similarly across cultures, a property known as measurement invariance. Measurement invariance evaluates the extent to which the items within an assessment instrument capture the same underlying construct either across distinct groups or time periods. Although researchers are often interested in cross-group or cross-time comparisons, it is not yet common to present evidence that those comparisons are based on comparable measures (9, 10). Moreover, psychological studies of measurement invariance comparing more than two groups are even less common. For example, from 126 invariance studies published between March 2013 and April 2014 in the *APA's PsycNet* database, Putnick and Bornstein (11) found that only 25% of invariance tests compared more than two groups.

Consider a simple example of potential consequences of measurement non-invariance. Suppose we wanted to compare Latinos and non-Latino English Speakers on distress by asking about *heart pounding, crying easily, headaches*, and *feeling lonely*. While these symptoms might be related to distress in both groups, the first two might be more easy for Latinos to admit than English Speakers for cultural reasons; moreover, in some samples there might be some instances of *heart pounding* and *crying easily* that are related to religious experiences rather than distress (12). As a result, if we compare Latinos and non-Latino English Speakers on a composite of these symptoms, the Latino group could incorrectly appear more distressed than the non-Latino White group because of symptom response styles, even though distress levels might actually be the same in both groups.

Since adequate statistical power to detect non-invariance depends upon the number of observations in each group being compared (13, 14), a major barrier to conducting invariance studies comparing more than two groups may be lack of adequate power. Invariance studies comparing many groups are thus particularly suitable for large-scale international surveys, which can include hundreds of thousands of observations. Cieciuch et al. (15), for example, evaluated invariance in a *values* scale using 274,447 respondents from 15 countries and six time periods (*average group size* = 3,049) from the European Social Survey (15). In contrast, psychological studies of invariance are often constrained by smaller samples. In the same review mentioned above, Putnick and Bornstein (11) also found a median total sample size of 725 observations. This would result in a relatively small group size (*N* ≈ 180) if, for example, the most prevalent US racial/ethnic groups were compared (English Speakers, Blacks, Latinos, and Asians). Given that racial/ethnic minorities have generally been underrepresented in randomized trials within psychiatry and psychology (16), sample sizes using data from randomized trials are in practice likely to be much smaller.

Despite sample size limitations, invariance testing of psychological constructs among racial/ethnic minorities is critical because health disparities are often measured using self-reported measures (17) and, to attend to linguistic diversity, these measures are increasingly being used in languages for which they were not originally developed and validated (18). Eliminating racial/ethnic health disparities has also become part of the national agenda (19). In addition, federal authorities have encouraged medical researchers to attend to diversity and inclusiveness in their work (3), creating numerous programs and policies intended to reduce disparities (20). However, racial/ethnic differences in self-reported measures cannot be used to infer disparities in theoretical attributes (e.g., late-life depression) and develop public health policies unless there is evidence that individuals from different groups responded similarly to the measures.

In the present study, we apply invariance tests to psychological measures in a sample of US minority older adults (60+ years old) using two common measures of anxiety and depression symptoms—the Generalized Anxiety Disorder 7-Item Scale [GAD-7 (21)] and the Hopkins Symptoms Checklist-25 [HSCL-25 (22, 23)]—and two measures of level of functioning—the Function Component of the Late Life Functioning and Disability Instrument [Late-Life FDI (24)] and the 12-item version of the World Health Organization Disability Assessment Schedule 2.0 [WHODAS 2.0 (25, 26)]. We examine the psychometric structure of the items that make up these measures when they were administered in four languages, using data from the Positive Minds-Strong Bodies (PMSB) randomized controlled trial (27). The PMSB trial was an evidence-based mental health and disability prevention intervention, which was delivered to a racially/ethnically diverse sample of 307 minority older adults in English (*N* = 66; 21.5%), Spanish (*N* = 138; 45.0%), Mandarin (*N* = 48; 15.6%), and Cantonese (*N* = 55; 17.9%).

Because the assessment instruments used to evaluate the effectiveness of PMSB were also applied in four languages based on participants' preference (27), invariance testing was performed comparing language groups. Almost all White and Black participants responded to the assessments in English (93.5 and 95.8%, respectively), almost all Latino participants responded in Spanish (95.6%) and almost all Asian participants responded in Mandarin or Cantonese (99.0%; see Table 1). Thus, analyzing language groups was almost equivalent to analyzing distinct races/ethnicities for Spanish, Mandarin and Cantonese speakers, but not for English speakers. However, in contrast with previous studies comparing racial/ethnic groups assessed in the same language [e.g., Vyas et al. (7)], the PMSB trial included minority older adults that would have otherwise been excluded (i.e., non-English speakers). To remain consistent with the design of the intervention (and because of very small samples within the White and Black racial groups), invariance tests were implemented comparing languages instead of race/ethnicity groups.

**Table 1:** Demographic baseline characteristics for the overall sample and by language.

	**Total Sample** ***N*** **=** **307**		**English** ***N*** **=** **66**		**Spanish** ***N*** **=** **138**		**Mandarin** ***N*** **=** **48**		**Cantonese** ***N*** **=** **55**		**χ^2^(df), *p***
	**N**	**%**	**N**	**%**	**N**	**%**	**N**	**%**	**N**	**%**	
**Age**											
60–64	21	6.84	3	4.55	16	11.59	1	2.08	1	1.82	χ^2^(6) = 38.72, *p* < 0.01
65–74	133	43.32	28	42.42	70	50.72	6	12.50	29	52.73	
75+	153	49.84	35	53.03	52	37.68	41	85.42	25	45.45	
**Gender**											
Male	59	19.22	7	10.61	29	21.01	9	18.75	14	25.45	χ^2^(3) = 4.82, *p* = 0.19
Female	248	80.78	59	89.39	109	78.99	39	81.25	41	74.55	
**Race**											
White	31	10.23	29	45.31	1	0.73	1	2.13	0	0.00	χ^2^(15) = 522.71, *p* < 0.01
Black	24	7.92	23	35.94	1	0.73	0	0.00	0	0.00	
American Indian	1	0.33	1	1.56	0	0.00	0	0.00	0	0.00	
Asian	102	33.66	1	1.56	0	0.00	46	97.87	55	100.00	
Latino	136	44.88	6	9.38	130	94.89	0	0.00	0	0.00	
Other	9	2.97	4	6.25	5	3.65	0	0.00	0	0.00	
**Education level**											
Less than high school	111	36.16	11	16.67	60	43.48	7	14.58	33	60.00	χ^2^(3) = 37.29, *p* < 0.01
High school or more	196	63.84	55	83.33	78	56.52	41	85.42	22	40.00	
**Place of birth**											
Outside of U.S	210	69.54	10	15.15	99	73.33	46	100.00	55	100.00	χ^2^(3) = 137.32, *p* < 0.01
U.S	92	30.46	56	84.85	36	26.67	0	0.00	0	0.00	
**Marital status**											
Married/cohabitating	96	31.27	7	10.61	31	22.46	30	62.50	28	50.91	χ^2^(9) = 65.64, *p* < 0.01
Divorced/separated	85	27.69	21	31.82	52	37.68	3	6.25	9	16.36	
Widowed	98	31.92	26	39.39	40	28.99	15	31.25	17	30.91	
Never married	28	9.12	12	18.18	15	10.87	0	0.00	1	1.82	
**Suicidal risk**^**a**^											
No	287	93.49	62	93.94	132	95.65	42	87.50	51	92.73	χ^2^(3) = 3.96, *p* = 0.27
Yes	20	6.51	4	6.06	6	4.35	6	12.50	4	7.27	
**Suicidal attempt**^**b**^											
No	288	99.65	63	98.44	126	100.00	46	100.00	53	100.00	χ^2^(3) = 3.53, *p* = 0.32
Yes	1	0.35	1	1.56	0	0.00	0	0.00	0	0.00	
**Any chronic condition**											
No	39	12.70	6	9.09	23	16.67	6	12.50	4	7.27	χ^2^(3) = 4.20, *p* = 0.24
Yes	268	87.30	60	90.91	115	83.33	42	87.50	51	92.73	
**Measures for invariance testing**	**M (SD) (range)**		**M (SD) (range)**		**M (SD) (range)**		**M (SD) (range)**		**M (SD) (range)**		**F(df1, df2)**, ***p***
GAD-7	6.0 (4.6) (0–21)		6.2 (4.3) (0–18)		6.8 (4.5) (0–20)		3.7 (4.0) (0–16)		5.6 (5.0) (0–21)		*F*_(3, 303)_ = 7.13, *p* < 0.01
HSCL-25	1.6 (0.4) (1–3)		1.6 (0.4) (1–3)		1.7 (0.5) (1–3)		1.5 (0.4) (1–2)		1.5 (0.5) (1–3)		*F*_(3, 306)_ = 4.82, *p* < 0.01
Late-life FDI	117.6 (26.1) (32–160)		112.0 (22.1) (65–153)		116.1 (28.5) (32–160)		120.8 (21.4) (82–158)		125.2 (26.2) (50–160)		*F*_(3, 306)_ = 3.43, *p* = 0.02
WHODAS 2.0	22.2 (7.5) (12–48)		21.9 (6.9) (13–41)		23.2 (8.0) (12–48)		20.8 (7.2) (12–39)		21.1 (6.8) (12–36)		*F*_(3, 306)_ = 1.89, *p* = 0.13

a *Suicidal risk includes participants who responded “yes” to either (1) feeling that life was not worth living, (2) wishing they were dead, and/or (3) having thoughts of taking their lives*.

b *Exclusion criteria included considering suicide/having a suicidal plan and/or suicide attempt during screening. One participant in the intervention group disclosed considering suicide/having a suicidal plan at baseline*.

## Methods

### Setting and Study Sample

Participants for the PMSB trial were recruited from clinical sites and community-based organizations in Massachusetts, New York, Florida and Puerto Rico between May 2015 and May 2018 (27). Research assistants approached potential participants in-person to administer a short screener after assessing their capacity to consent. A full screener was administered if participants were 60+ years old and spoke either English, Spanish, Mandarin or Cantonese. Eligible participants had screening measures indicative of mild to severe depressive or anxiety symptoms—scored five or more on either the Patient Health Questionnaire (28), the Geriatric Depression Scale (29) or the GAD-7 (21)—and reported some degree of mobility limitations—Short Physical Performance Battery scores between three and 11 (30). Participants disclosing serious suicide plans or attempts were referred to emergency health services and rescreened 30 days after; they were eligible if found to be non-suicidal, and ineligible otherwise.

From 1,057 individuals whom were fully screened, 307 were eligible and agreed to participate—and then randomized to the intervention or control groups and scheduled for a baseline interview (27). Additional interviews were administered two, six and 12 months after baseline using participants' preferred language (66 English, 138 Spanish, 48 Mandarin, and 55 Cantonese). For the present study, we used data from the baseline assessment (before any of the 307 eligible participants received the intervention). All assessments were structured in-person interviews by trained bilingual interviewers. The Institutional Review Boards of Massachusetts General Hospital/Partners HealthCare and New York University approved the study protocol.

### Measures

#### Anxiety and Depression

##### GAD-7

The GAD-7 is a 7-item self-reported measure of probable cases of Generalized Anxiety Disorder (21). Respondents are asked how often, during the last 2 weeks, they were bothered by each symptom, with responses rated on a 4-point scale (0 = *not at all* and 3 = *nearly every day*). Total scores are calculated summing all items (range: 0–21), and higher scores represent worse symptoms. Previous studies in the general population have found a 1-factor model to be the preferable solution (31).

##### HSCL-25

The HSCL-25 is a 25-item screener of mood symptoms—ten anxiety symptoms and 15 depressive symptoms (22, 23). Respondents are asked how much they were bothered by each symptom in the last 4 weeks, with responses rated on a 4-point scale (1 = *not at all* and 4 = *extremely*). Total scores are computed averaging all items (range: 1–4), and higher scores represent worse symptoms. A 2-factor model comprising symptoms specific to anxiety and symptoms specific to depression has been found to be the preferable solution (32, 33).

#### Level of Functioning

##### Late-Life FDI

The Late-Life FDI is a 32-item self-reported measure assessing difficulty performing daily physical activities in older adults (24). Respondents are asked about difficulties performing an activity without help from someone else or the use of assisted devices, with responses rated on a 5-point scale (1 = *cannot do* and 5 = *none*). Total scores are calculated summing all items (range: 32–160), and scores approaching 32 indicate poor ability. A 3-factor solution has been found to explain most of the variance (24), with seven items representing *upper extremity functioning*, 14 items representing *basic lower extremity functioning*, and 11 items representing *advanced lower extremity functioning*.

##### WHODAS 2.0

The 12-item version of the WHODAS 2.0 is a brief generic instrument assessing level of functioning in six domains of life: Cognition, mobility, self-care, getting along, life activities, and participation (25, 26). Respondents are asked about functioning difficulties experienced in the last 30 days, with responses rated on a 5-point scale (1 = *none* and 5 = *extreme or cannot do*). Final scores are calculated summing all items (range: 12–60), with higher scores representing more difficulties.

### Assessment Languages

Most measures included in the present study had been previously translated and psychometrically evaluated for use among Spanish, Mandarin, and Cantonese speakers. Although Mandarin and Cantonese translation-equivalents are orthographically identical—in fact, the Chinese Academy of Social Sciences refers to Mandarin and Cantonese as two dialects of the same language (34)—they have many characteristics associated with distinct languages, and their spoken forms are mutually unintelligible (35, 36). Since all measures were collected via structured interviews by trained bilingual interviewers, in practice these measures were administered in four different languages, even though the written versions were the same in Mandarin and Cantonese.

Translations for Spanish speakers were available for the GAD-7 (37) and the WHODAS 2.0 (38, 39). Translations for Mandarin and Cantonese speakers were available for the GAD-7 (40), the HSCL-25 (41) and the WHODAS 2.0 (38, 39). Other translations (i.e., the HSCL-25 for Spanish speakers and the Late-Life FDI for Spanish, Mandarin, and Cantonese speakers) were performed using the English version, first by professional translators and then by bilingual PMSB staff. These translations were thoroughly reviewed and edited by supervising PMSB staff and back translated into English. A multicultural committee of clinicians and staff at partner agencies was convened afterwards to compare translations and back translations. When the back translations revealed ambiguities, a multinational panel of researchers knowledgeable about the measures were engaged to resolve them (27).

### Statistical Analysis

We began by describing baseline demographic and clinical characteristics (age, gender, race/ethnicity, education, birthplace, marital status, suicidal behaviors, and chronic conditions) for the total sample and by language, using χ^2^ tests to assess significant group differences. We also presented descriptive statistics (means, standard deviations, and range) for our two measures of anxiety and depression (GAD-7 and HSCL-25) and our two measures of level of functioning (Late-Life FDI and WHODAS 2.0) in the total sample and by language, using two-tailed *F*-tests to assess significant group differences. We then tested measurement invariance using multiple group confirmatory factor analysis [CFA (42)]. In CFA, item response variation for each scale is modeled as a reflection of a latent factor representing a theoretical construct. In factor analysis terminology, we say the items load on a single factor.

#### Measurement Invariance Models

Based on a sequence of nested models, we tested three different levels of equivalence (43): Configural (equivalence of model form), metric (equivalence of factor loadings), and scalar (equivalence of item means). Since final scores of all analyzed measures are commonly used as a continuous scale, we treated the observed item responses as continuous variables. Additionally, we fitted separate models to each subscale of the HSCL-25 and the Late-Life FDI (i.e., anxiety and depression subscales for the HSCL-25, and *upper extremity functioning, basic lower extremity functioning*, and *advanced lower extremity functioning* for the Late-Life FDI) to make the one factor solution more plausible. Models were estimated using the robust maximum likelihood mean and variance adjusted estimator in Mplus 7.4 (44). To concretely illustrate each step, we focused on an example using the GAD-7 to compare anxiety symptoms between English, Spanish, Mandarin and Cantonese speakers. In this particular case, anxiety would be measured through seven continuously distributed items (e.g., *feeling nervous, worrying too much*) that load onto a latent factor that represents anxiety.

##### Configural invariance

Configural invariance assesses whether the unobserved factor (in our example the latent factor of anxiety) was related to item responses similarly across languages; that is, whether the factor structure is the same. Invariance at this level means that the basic organization of the latent construct is the same in all four languages, i.e., that the GAD-7 items load onto the same anxiety latent factor in all four languages. It is tested by evaluating overall model fit according to the criteria described below.

##### Metric invariance

Metric invariance assesses whether item factor loadings are similar across languages, suggesting that the latent variable is related to specific item translations to a similar degree. This model is nested within the configural model because it has the same structure but imposes equality constraints on the factor loadings. In our example, the loadings of the GAD-7 items (i.e., the loadings of the seven items on the anxiety construct) are set to be equivalent across language groups. Metric invariance holds if model fit is not worse compared to the configural model.

##### Scalar invariance

Scalar invariance assesses whether the item means are equivalent across languages after adjusting for possible group differences in the level of the latent variable (i.e., anxiety in our example). This model is nested within the metric model because it has the same structure but imposes equality constraints on the item intercepts, which reflect the adjusted item means. In our example, the item intercepts (means) of the seven items that load onto the anxiety construct are set to be equivalent across language groups. Scalar invariance holds if model fit is not worse compared to the metric model.

##### Partial invariance

If either metric or scalar invariance did not hold, we applied the concept of partial invariance (45) by identifying and setting free the factor loadings (partial metric invariance) and intercepts (partial scalar invariance) responsible for non-invariance. Metric non-invariance means that at least one loading is not equivalent across languages. In our example, non-invariance of a loading related to *worrying too much* would mean that this item is either more or less closely related to the latent construct of anxiety in one language than in the others. Scalar non-invariance indicates that at least one item intercept (mean) differs across languages. In our example, non-invariance of an item intercept for *worrying too much* would mean that speakers from one language are bothered either more or less by this symptom, but that is not related to increased or decreased levels of anxiety in that language group. Although it is recommended that a majority of the items be invariant (46), partial scalar invariance allows cross-language latent (not observed) mean differences to remain meaningful, provided that at least two of the items are invariant (47). In addition, the summed—or averaged—item responses of the invariant items can be used to compare groups (48).

When only partial scalar invariance was supported (such that only item responses from invariant items can be used to compare groups), we calculated an approximate measure of bias—and its 95% confidence interval—for each pair of languages. We defined *Bias* = Δ_I+N_ – Δ_I_, where Δ_I+N_ is the mean difference in invariant plus non-invariant items and Δ_I_ is the mean difference in invariant items only. Consistent with previous literature, we considered |*Bias*| < 0.05 indicative of trivial bias, 0.05 ≤ |*Bias*| ≤ 0.10 indicative of moderate bias, and |*Bias*| > 0.10 indicative of substantial bias (49, 50).

#### Fit of Measurement Invariance Models

We assessed model fit using the Comparative Fit Index (CFI), the Tucker-Lewis Index (TLI), and the Root Mean Squared Error of Approximation (RMSEA). CFI and TLI values above 0.90 and 0.95 are considered adequate and good, respectively; RMSEA values below 0.08 and 0.05 are considered adequate and good, respectively (51, 52). Configural invariance held if configural model fit was either good or adequate. To compare fit between nested models (i.e., metric invariance model vs. configural invariance model and scalar invariance model vs. metric invariance model), we used the χ^2^ difference test (Δχ^2^), the difference in the CFI (ΔCFI) and the difference in the RMSEA (ΔRMSEA). Fit of the nested model was not worse compared to the less restricted model if either Δχ^2^ was not significant at the α = 0.05 level (53) or ΔCFI ≤ −0.01 (14) and ΔRMSEA ≤ 0.01 (54). That is, fit of the metric invariance model was not worse compared to configural invariance model (i.e., metric invariance held) if either Δχ^2^ was not significant at the α = 0.05 level (*p* < 0.05) or ΔCFI ≤ −0.01 and ΔRMSEA ≤ 0.01. Analogously, fit of the scalar invariance model was not worse compared to the metric invariance model (i.e., scalar invariance held) if either Δχ^2^ was not significant at the α = 0.05 level (*p* < 0.05) or ΔCFI ≤ −0.01 and ΔRMSEA ≤ 0.01. The same model comparison fit criteria applied to partial invariance models.

#### Power to Detect Non-invariance

The number of observations included in measurement invariance tests is known to influence the power to detect non-invariance (13, 14). However, when it comes to invariance testing large samples are not necessarily the rule of thumb: Power to reject the hypothesis of invariance using Δχ^2^ increases as the sample size increases, which may lead to the erroneous conclusion that there is measurement non-invariance in large samples. Measurement invariance tests have thus shifted toward changes in alternative fit indices (such as ΔCFI and ΔRMSEA) because they are less sensitive to variations in sample size (14). To increase the likelihood that our sample size would not be associated with the level of measurement invariance achieved, we chose to use ΔCFI and ΔRMSEA (in addition to Δχ^2^) because these two model fit indices are less sensitive to sample size.

## Results

Table 1 presents the distribution of demographic and clinical characteristics in the total sample and by language, including χ^2^ tests for significant group differences. Most participants were 75+ years old (49.8%), female (80.8%), Latino (44.9%), widowed (31.9%), had a high school degree or more (63.8%) and at least one chronic condition (87.3%). English speakers were more likely to self-identify as either White (45.3%) or Black (35.9%) and to be US born (84.9%). Spanish speakers were younger, less educated, and more likely to self-identify as Latino (94.9%) and to be foreign born (73.3%). Mandarin speakers were all foreign born and more likely to self-identify as Asian (97.9%) and to be married or cohabitating (62.5%). All Cantonese speakers self-identified as Asian and were foreign born.

In Table 1 we also present the distribution of the four measures used to test measurement invariance in the total sample and by language. Compared to English speakers, Mandarin speakers reported lower anxiety symptoms per the GAD-7 (*p* < 0.01) but Spanish and Cantonese speakers reported the same level of anxiety (*p* = 0.31 and *p* = 0.50, respectively). Regarding mood symptoms, Spanish speakers had higher HSCL-25 scores than English speakers (*p* = 0.03), while both Mandarin and Cantonese speakers presented the same level of mood symptoms than English speakers (*p* = 0.22 and *p* = 0.64, respectively). Level of functioning as measured by the Late-Life FDI was the same among Spanish speakers compared to English speakers (*p* = 0.29), but Mandarin and Cantonese speakers had both higher levels of functioning than English speakers (*p* = 0.03 and *p* < 0.01, respectively). There were no significant differences across language groups in level of functioning as measured by the WHODAS 2.0.

### Measurement Invariance: GAD-7

Table 2 shows multiple group CFA results. A summary of the items that were found to have some degree of non-invariance is presented in Table 3. Regarding the GAD-7, configural model fit was adequate, indicating that the latent construct was conceptualized similarly across languages. There was evidence of similarity of factor loadings (metric invariance) but not of item intercepts. We investigated the source of scalar non-invariance by sequentially releasing (in a backward approach) item intercepts constraints and retesting the model. Partial scalar invariance was achieved after releasing the intercepts of two items. Adjusting for the latent variable, English and Spanish speakers reported being bothered more often by the symptom *worry too much* (i.e., higher item means) whereas Spanish and Mandarin speakers reported being bothered more often by the symptom *restless/hard to sit still* compared to respondents in other languages with the same level of anxiety.

**Table 2:** Measurement invariance testing of PMSB outcome measures across four language groups.

**Model**	**Model fit statistics**				**Measurement invariance test statistics**				
	**χ^2^ (*df*)**	**CFI**	**TLI**	**RMSEA (90% CI)**	**Δχ^2^ (Δ*df*)**	**Δ*p***	**ΔCFI**	**ΔRMSEA**	**Decision**
**GAD-7**									
1. Configural	72.04 (56)	0.945	0.917	0.061 (0.000, 0.099)					
2. Metric (vs. 1)	90.06 (74)	0.945	0.937	0.053 (0.000, 0.089)	18.07 (18)	0.451	0.000	−0.008	Accept
3. Scalar (vs. 2)	122.38 (92)	0.896	0.905	0.066 (0.028, 0.095)	45.44 (18)	<0.01	−0.049	0.013	Reject
3a. Partial scalar (vs. 2)	106.25 (86)	0.930	0.932	0.056 (0.000, 0.088)	19.71 (12)	0.073	−0.015	0.003	Accept
**HSCL-25**									
Anxiety subscale^a^									
1. Configural	171.72 (140)	0.925	0.904	0.054 (0.016, 0.080)					
2. Metric (vs. 1)	195.71 (167)	0.932	0.927	0.047 (0.000, 0.073)	24.66 (27)	0.594	0.007	−0.007	Accept
3. Scalar (vs. 2)	251.89 (194)	0.864	0.873	0.062 (0.038, 0.083)	96.97 (27)	<0.01	−0.068	0.015	Reject
3a. Partial scalar (vs. 2)	213.29 (182)	0.926	0.927	0.047 (0.000, 0.072)	21.05 (15)	0.135	−0.006	0.000	Accept
Depression subscale^b^									
1. Configural	231.54 (192)	0.941	0.918	0.052 (0.019, 0.075)					
2. Metric (vs. 1)	265.93 (225)	0.938	0.928	0.049 (0.016, 0.071)	40.21 (33)	0.181	−0.003	−0.003	Accept
3. Scalar (vs. 2)	343.09 (258)	0.872	0.869	0.066 (0.046, 0.084)	136.71 (33)	<0.01	−0.066	0.017	Reject
3a. Partial scalar (vs. 2)	299.81 (252)	0.928	0.925	0.050 (0.021, 0.070)	43.60 (27)	0.023	−0.010	0.001	Accept
**Late-Life FDI**									
Upper extremity^c^									
1. Configural	51.63 (36)	0.941	0.902	0.076 (0.013, 0.120)					
2. Metric (vs. 1)	59.79 (51)	0.967	0.961	0.048 (0.000, 0.092)	11.28 (15)	0.733	0.026	−0.028	Accept
3. Scalar (vs. 2)	96.63 (66)	0.885	0.896	0.079 (0.041, 0.111)	57.15 (15)	<0.01	−0.082	0.031	Reject
3a. Partial scalar (vs. 2)	66.05 (57)	0.966	0.964	0.046 (0.000, 0.088)	6.77 (6)	0.343	−0.001	−0.002	Accept
Basic lower extremity^d^									
1. Configural	265.01 (216)	0.935	0.921	0.055 (0.027, 0.076)					
2. Metric (vs. 1)	306.07 (249)	0.925	0.920	0.055 (0.030, 0.075)	47.93 (33)	0.045	−0.010	0.000	Accept
3. Scalar (vs. 2)	373.57 (282)	0.879	0.887	0.065 (0.046, 0.083)	114.94 (33)	<0.01	−0.046	0.010	Reject
3a. Partial scalar (vs. 2)	330.50 (267)	0.916	0.917	0.056 (0.031, 0.074)	33.61 (18)	0.014	−0.009	0.001	Accept
Advanced lower extremity^e^									
1. Configural	252.08 (176)	0.925	0.906	0.077 (0.054, 0.098)					
2. Metric (vs. 1)	280.13 (203)	0.924	0.917	0.072 (0.050, 0.092)	26.77 (27)	0.476	−0.001	−0.005	Accept
3. Scalar (vs. 2)	367.80 (230)	0.864	0.870	0.091 (0.073, 0.108)	133.09 (27)	<0.01	−0.060	0.019	Reject
3a. Partial scalar (vs. 2)	305.30 (218)	0.914	0.913	0.074 (0.053, 0.093)	34.36 (15)	<0.01	−0.010	0.002	Accept
**WHODAS 2.0**									
1. Configural	239.10 (192)	0.910	0.876	0.058 (0.029, 0.080)					
2. Metric (vs. 1)	288.94 (225)	0.877	0.856	0.062 (0.032, 0.082)	61.98 (33)	<0.01	−0.033	0.004	Reject
2a. Partial metric (vs. 1)	271.48 (219)	0.899	0.879	0.057 (0.027, 0.077)	39.93 (27)	0.052	−0.011	−0.001	Accept
3. Scalar (vs. 2a)	316.04 (246)	0.866	0.856	0.062 (0.039, 0.081)	64.80 (27)	<0.01	−0.033	0.005	Reject
3a. Partial scalar (vs. 2a)	291.99 (237)	0.894	0.882	0.056 (0.030, 0.077)	24.05 (18)	0.153	−0.005	−0.001	Accept

a *Includes the first 10 items of the HSCL-25*.

b *Includes items 11-15 of the HSCL-25*.

c *Includes items 1, 5, 6, 13, 16, and 17 of the Late-Lile FDI*.

d *Includes items 2, 10, 11, 14, 15, 18, 21, 22, 23, 26, 28, and 31 of the Late-Life FDI*.

e *Includes items 4, 7, 8, 9, 19, 20, 24, 27, 29, 30, and 32 of the Late-Life FDI*.

**Table 3:** Summary of non-invariant items.

**Item**	**Type of non-invariance**	**Description**
**GAD-7**		
GAD3. Worry too much	Scalar (item means)	English and Spanish speakers were bothered more often by this symptom in the last 2 weeks compared to Mandarin and Cantonese, but that was not related to increased levels of anxiety.
GAD5. Restless/hard to sit still	Scalar (item means)	Spanish and Mandarin speakers were bothered more often by this symptom in the last 2 weeks compared to English and Cantonese, but that was not related to increased levels of anxiety.
**HSCL-25**		
Anxiety subscale		
HSCL3: Faintness, dizziness, or weakness HSCL7: Tense or keyed up HSCL8: Headaches HSCL9: Spells of terror or panic	Scalar (item means)	All these items appeared to be related to *somatic symptoms* of anxiety. Spanish speakers were bothered less by all of these *somatic symptoms* in the last 4 weeks compared to the other three languages, but that was not related to lower levels of anxiety.
Depression subscale		
HSCL13: Crying easily	Insignificant factor loading	Unrelated to the underlying construct among English speakers.
HSCL14: No sexual interest or pleasure	Insignificant factor loading	Unrelated to the underlying construct among Mandarin and Cantonese speakers.
HSCL20: Thoughts of ending your life	Insignificant factor loading	Unrelated to the underlying construct in all languages.
HSCL22: Worry too much	Scalar (item means)	English and Spanish speakers were bothered more by this symptom in the last 4 weeks compared to Mandarin and Cantonese, but that was not related to increased levels of depression.
HSCL23: No interest in things	Scalar (item means)	Mandarin and Cantonese speakers were bothered more by this symptom in the last 4 weeks compared to English and Spanish, but that was not related to increased levels of depression.
**Late-Life FDI**		
Upper extremity subscale		
LLF1. Unscrew lid	Scalar (item means)	Mandarin speakers had more difficulty performing this activity on a daily basis compared to the other three languages, but that was not related to decreased upper extremity functioning.
LLF3. On/off trousers	Insignificant factor loading	Unrelated to the underlying construct among Mandarin speakers.
LLF13. Reach behind back	Scalar (item means)	Mandarin and Cantonese speakers had less difficulty performing this activity on a daily basis compared to English and Spanish, but that was not related to increased upper extremity functioning.
LLF16. Remove wrapping	Scalar (item means)	Mandarin speakers had more difficulty performing this activity on a daily basis compared to the other three languages, but that was not related to decreased upper extremity functioning.
Basic lower extremity subscale		
LLF12. On/off coat or jacket	Insignificant factor loading	Unrelated to the underlying construct among Mandarin speakers.
LLF25. Bend over to pick up clothes		
LLF15. Open heavy door, outside LLF21. Pick up chair and move it to clean LLF22. Use step stool LLF26. Walk around one floor of home LLF28. Wash dishes while standing	Scalar (item means)	Mandarin and Cantonese speakers had more difficulty performing all of these activities on a daily basis compared to English and Spanish, but that was not related to decreased basic lower extremity functioning.
Advanced lower extremity subscale		
LLF20. 3 flights of stairs inside, handrail LLF29. Walk several blocks LLF30. Take a 1-mile walk, no rest LLF32. Walk on a slippery surface	Scalar (item means)	Mandarin/Cantonese speakers had more difficulty performing these activities on a daily basis compared to English/Spanish, but that was not related to decreased advanced lower extremity functioning.
**WHODAS 2.0**		
WHO3. Learn new task	Metric (factor loadings)	*Learning a new task* was more related to the cognition domain of the WHODAS 2.0 in Mandarin/Cantonese compared to English/Spanish.
WHO7. Walk 0.6+ miles	Scalar (item means)	Mandarin/Cantonese speakers had more difficulty performing this activity in the last 30 days compared to English/Spanish, but that was not related to decreased functioning.
WHO9. Get dressed	Metric (factor loadings)	*Getting dressed* was less related to the self-care domain of the WHODAS 2.0 in Mandarin/Cantonese compared to English/Spanish.
WHO11. Maintaining a friendship	Scalar (item means)	Mandarin speakers had more difficulty performing this activity in the last 30 days compared to the other three languages, but that was not related to decreased levels of functioning.
WHO12. Day-to-day work/school	Scalar (item means)	Spanish speakers had less difficulty performing this activity in the last 30 days compared to the other three languages, but that was not related to increased levels of functioning.

### Measurement Invariance: HSCL-25

#### Anxiety Subscale

In the anxiety subscale, configural model fit was adequate and fit of the metric model was not worse compared to the configural model, but fit of the scalar model was worse compared to the metric model. Partial scalar invariance was achieved after freeing the intercepts of four items related to somatic symptoms of anxiety (see Table 3). After adjusting for the latent variable, Spanish speakers reported being bothered less by these somatic symptoms (i.e., lower item means) compared to English, Mandarin and Cantonese speakers with the same level of anxiety.

#### Depression Subscale

Configural model fit was inadequate in the depression subscale (CFI = 0.817, TLI = 0.787, RMSEA = 0.068), and this model indicated that three items were unrelated to the underlying construct (see Table 3). Model fit improved after removing these items but was still inadequate (CFI = 0.877, TLI = 0.850, RMSEA = 0.070). In exploratory factor analysis (EFA) we found a very strong general factor (first to second eigenvalue ratio of 5.55–1.02) with a second factor clustering the four items related to somatic symptoms of depression: *Low energy/slowed down, poor appetite, no interest in things* and *feeling everything is an effort*. We modeled this clustering using a bifactor model, with one general depression factor and one *somatic-symptoms* factor uncorrelated with the general factor. This strategy isolates item response variation unaccounted for by the general depression factor. Configural model fit became adequate and there was evidence of metric invariance but not of scalar invariance. Partial scalar invariance was achieved by freeing the intercepts of two items: After adjusting for the latent variable, English and Spanish speakers reported being bothered more by the symptom *worry too much* whereas Mandarin and Cantonese speakers reported being bothered more by the symptom *no interest in things* compared to respondents in other languages with the same level of depression.

### Measurement Invariance: Late-Life FDI

#### Upper Extremity Functioning Factor

Configural model fit for this factor was inadequate (CFI = 0.900, TLI = 0.849, RMSEA = 0.089), and this model indicated that one item was unrelated to the underlying construct in Mandarin. After removing this item, fit of the configural model improved and became adequate. There was also evidence of metric invariance, and of partial scalar invariance after freeing the intercepts of three items. Adjusting for the latent variable, Mandarin speakers reported more difficulty performing the activities *unscrew lid* and *remove wrapping* whereas Mandarin and Cantonese speakers reported less difficulty performing the activity *reaching behind back* compared to respondents in other languages with the same level of functioning.

#### Basic Lower Extremity Functioning Factor

Configural model fit for this factor was adequate per the CFI and RMSEA but not per the TLI (CFI = 0.915, TLI = 0.900, RMSEA = 0.056), and this model suggested that two items were unrelated to the underlying construct in Mandarin. Configural model fit was adequate after removing these two items, and there was evidence of equality of factor loadings but not of equality of item intercepts. Partial scalar invariance was achieved after freeing the intercepts of the five items. Adjusting for the latent variable, Mandarin and Cantonese speakers reported more difficulty performing the activities listed on these items (see Table 3) compared to English and Spanish speakers with the same level of functioning.

#### Advanced Lower Extremity Functioning Factor

Configural model fit for this factors was inadequate (CFI = 0.917, TLI = 0.896, RMSEA = 0.081). In EFA we found a very strong general factor (first to second eigenvalue ratio of 5.95–0.83) but two items related to *walking* clustered in a separate factor. We modeled this clustering using a bifactor model with one general advanced lower extremity factor and one *walking-symptoms* factor uncorrelated with the general factor. Configural model fit became adequate, fit of the metric model was not worse compared to the configural model, and partial scalar invariance held after freeing the intercepts of four items. Adjusting for the latent variable, Mandarin and Cantonese speakers reported more difficulty performing the activities listed on these items (see Table 3) compared to English and Spanish speakers with the same level of functioning.

### Measurement Invariance: WHODAS 2.0

Configural model fit for the WHODAS 2.0 was inadequate (CFI = 0.739, TLI = 0.682, RMSEA = 0.092). In EFA, we found a very strong general factor (first to second to third eigenvalue ratio of 4.50 to 1.31 to 1.07), but four items clustered in two separate factors corresponding to two of the six disability domains: Mobility (*stand for 30*+ *min* and *walk 0.6*+ *miles*) and self-care (*wash whole body* and *get dressed*). We modeled this clustering using a bifactor model, with one general disability factor and six domain specific factors uncorrelated with the general factor. Configural model fit improved and although the TLI still indicated inadequate fit, we continued invariance testing using this bifactor model. Only partial metric and partial scalar invariance were achieved. Partial metric invariance held after allowing two factor loadings to be freely estimated, while partial scalar invariance held after allowing three item intercepts to be freely estimated. Compared to English and Spanish speakers, *learn new task* was more related to the cognition domain and *get dressed* was less related to the self-care domain among Mandarin and Cantonese speakers. In addition, after adjusting for the latent variable, Mandarin speakers reported more difficulty with *walk 0.6*+ *miles* and *maintaining a friendship* while Spanish speakers reported less difficulty with *day-to-day school/work* compared to respondents in other languages with the same level of functioning.

### Bias From Removing Non-Invariant Items in Cross-Language Comparisons

Since only partial scalar invariance was supported for all measures, we calculated the bias from removing non-invariant items in cross-language comparisons (Table 4). Bias was either trivial or moderate, and there was significant substantial bias in only three out of 42 pairwise comparisons: Removing non-invariant items would underestimate (*Bias* > 0) mean differences between English and Spanish speakers in the anxiety subscale of the HSCL-25 (*effect size* = 0.27), mean differences between Spanish and Mandarin speakers in upper extremity functioning (*effect size* = 0.39), and mean differences between English and Mandarin speakers in basic lower extremity functioning (*effect size* = 0.30).

**Table 4:** Estimated bias in cross-language observed mean differences.

	**English vs.** **Spanish**	**English vs.** **Mandarin**	**English vs.** **Cantonese**	**Spanish vs.** **Mandarin**	**Spanish vs.** **Cantonese**	**Mandarin vs.** **Cantonese**
**GAD-7**	−0.045 (−0.229, 0.139)	−0.015 (−0.234, 0.205)	0.032 (−0.213, 0.276)	0.030 (−0.163, 0.224)	0.076 (−0.145, 0.297)	0.046 (−0.205, 0.298)
**HSCL-25**						
Anxiety subscale^a^	0.171* (0.005, 0.336)	0.030 (−0.161, 0.222)	0.030 (−0.164, 0.225)	−0.140 (−0.329, 0.048)	−0.140 (−0.332, 0.052)	0.000 (−0.214, 0.215)
Depression subscale^b^	0.021 (−0.189, 0.232)	0.045 (−0.215, 0.304)	0.024 (−0.234, 0.281)	0.024 (−0.199, 0.247)	0.003 (−0.218, 0.223)	−0.021 (−0.289, 0.247)
**Late-Life FDI**						
Upper extremity^c^	−0.116 (−0.331, 0.098)	0.131 (−0.120, 0.383)	−0.169 (−0.453, 0.116)	0.247* (0.005, 0.490)	−0.053 (−0.329, 0.224)	−0.300 (−0.606, 0.006)
Basic lower extremity^d^	0.093 (−0.147, 0.333)	0.291* (0.027, 0.554)	0.183 (−0.104, 0.470)	0.198 (−0.045, 0.440)	0.090 (−0.178, 0.357)	−0.108 (−0.397, 0.181)
Advanced lower extremity^e^	−0.063 (−0.357, 0.231)	0.173 (−0.192, 0.537)	0.176 (−0.178, 0.529)	0.236 (−0.100, 0.571)	0.239 (−0.085, 0.562)	0.003 (−0.386, 0.392)
**WHODAS 2.0**	0.061 (−0.117, 0.239)	0.086 (−0.133, 0.305)	0.000 (−0.205, 0.205)	0.025 (−0.177, 0.227)	−0.061 (−0.248, 0.126)	−0.086 (−0.313, 0.140)

95% confidence intervals in brackets; * p < 0.05.

a *Includes the first 10 items of the HSCL-25*.

b *Includes items 11-15 of the HSCL-25*.

c *Includes items 1, 5, 6, 13, 16, and 17 of the Late-Life FDI*.

d *Includes items 2, 10, 11, 14, 15, 18, 21, 22, 23, 26, 28, and 31 of the Late-Life FDI*.

e *Includes items 4, 7, 8, 9, 19, 20, 24, 27, 29, 30, and 32 of the Late-Life FDI*.

## Discussion

### Overview

Using a racially/ethnically diverse sample of US minority older adults, we applied invariance tests to common measures of anxiety, depression and level of functioning comparing four languages: English, Spanish, Mandarin, and Cantonese. We found that the underlying theoretical constructs were conceptualized comparably in all four languages, and that item response data had a similar psychometric structure across groups. However, item-means were only partially equivalent after adjusting for possible group differences in the level of the latent variable (i.e., speakers from certain language groups were bothered more or less often by some symptoms, but that was not related to increased or decreased levels of the theoretical construct). Since only item responses from invariant items can be used to compare language groups, we calculated the bias from omitting items that appeared to function differently, and found that omitting these items did not introduce substantial bias in cross-language comparisons. Nevertheless, we identified a non-negligible number of items that may require further study before their use to compare symptoms of anxiety, depression and level of functioning among linguistically diverse older adult populations: Two out of seven items in the GAD-7; nine out of 25 items in the HSCL-25; 15 out of 32 items in the Late-Life FDI; and five out of 12 items in the WHODAS 2.0.

### Anxiety and Depression

English and Spanish speakers reported more worry symptoms in both the GAD-7 and the depression subscale of the HSCL-25 for reasons unrelated to anxiety and depression, which is consistent with prior literature comparing expression of psychological distress across cultures. In a diverse cohort of cancer patients 21–84 years old, Teresi et al. (55) found that Latinos, Blacks and Spanish speakers were posited to express greater worry in the Patient Reported Outcomes Measurement Information System (PROMIS) Anxiety item bank (55). Similarly, Varela et al. (56) found that US Hispanic youth reported more worry symptoms than US European American youth in the Revised Children's Manifest Anxiety Scale (56). Since our study sample was made of older adults 60+ years old, our findings suggest then that Latinos (most of whom were assessed in Spanish) are more likely to express symptoms of worry for reasons unrelated to anxiety throughout their lifespan, and that measuring anxiety via worry symptoms among Latinos and Spanish speakers might not be warranted.

We also found that Spanish speakers (94.9% of whom self-identified as Latino) and Mandarin speakers (97.9% of whom self-identified as Asian) reported feeling more restless in the GAD-7 for reasons unrelated to anxiety. In the same cohort of cancer patients 21–84 years old, Teresi et al. (55) found that Latinos and Asians showed a higher probability of reporting feeling anxious in the PROMIS Anxiety item bank (55). Our results are thus consistent with this previous finding since restlessness is one of the most commonly reported symptoms of feeling anxious, highlighting the need to carefully examine whether feelings of restlessness are a true indicator of anxiety symptoms among Spanish and Mandarin speakers.

We encountered that Spanish speakers reported being bothered less on several somatic symptoms items of the HSCL-25 (*faintness, dizziness or weakness*; *tense or keyed up*; *headaches*; and *spells of terror or panic*) for reasons unrelated to anxiety. There is a common notion that Latinos report more somatic symptoms of psychological distress than English Speakers (57, 58), but recent evidence also suggests that Latino older adults might not somaticize their psychological distress. Letamendi et al. (59), for example, found that while many older Mexican-Americans experience clinically significant criteria for anxiety and depression, endorsement of physical symptoms of psychological distress was very low in the Brief Symptom Inventory-18 Spanish Version, a widely used tool to assess symptoms of anxiety, depression and somatization (59). Similarly, Teresi and Golden (60) found that some of the somatic symptoms of the SHORT-Comprehensive and Assessment and Referral Evaluation Depression scale were relatively less severe indicators of depression for Latinos than for English Speakers (60). It is possible then that Latinos report either more or less somatic symptoms for reasons unrelated to psychological distress at different timepoints througout their lives. Regardless, it seems to be the case that Latinos tend to express somatic symtpoms of psychopathology differently compared to other cultures, and these differences in somatization could be primarily cultural rather than linguistic.

We also found that Mandarin and Cantonese speakers, most of whom self-identified as Asian, reported being bothered more by the symptom *no interest in things* in the HSCL-25 for reasons unrelated to depression. This finding is consistent with previous work by Zhao et al. (61) whom found that *loss of interest* items in five depression measures had low discriminating power to distinguish Chinese patients with varied levels of depression, and that these items were only associated with moderate but not severe depressive symtpoms (61). Prior research has argued that compared to Western cultures, Chinese older adults are more likely to place greater emphasis on meeting sociocultural demands—possibly because they perceive future time as more limited—and to adjust personal goals to make them consistent with their cultural values (62). It is possible then that Mandarin and Cantonese speakers interpreted *no interest in things* as a symtpom related to their own self, so they reported being bothered more by this symptom to reflect a shift toward prioritizing cultural values over personal goals, and not for reasons related to depressive symptoms.

Finally, we found that three items were unrelated to the underlying construct in the depression subscale of the HSCL-25: *Crying easily* in English, *no sexual interest or pleasure* in Mandarin and Cantonese, and *thoughts of ending your life* in all languages. Thus, we dropped these items and tested invariance using 12-items instead of 15. Regarding *crying easily* and *thoughts of ending your life*, we believe this result might be associated with specific characteristics of our sample. Almost 90% of the English speakers were female, who have been consistently found to report crying more frequently for reasons unrelated to psychological distress (63). Crying has also been found to be weakly associated with depression among US older adults (64). As noted in the section Methods, participants disclosing serious suicide plans or attempts were ineligible to participate in the study, and this was most likely the reason why *thoughts of ending your life* was unrelated to the underlying construct in all languages. In regard to *no sexual interest or pleasure*, our results support the claim that Asian populations are more reluctant to discuss sexual topics (65) and that they also suppress the expression of emotional/affective symptoms (66).

### Level of Functioning

Mandarin and Cantonese speakers reported more difficulties performing physical activities in both the Late-Life FDI and the WHODAS 2.0 for reasons unrelated to their levels of functioning. We observed this result for basic/moderate tasks like *unscrewing a lid, removing wrapping* or *washing dishes* and for more strenuous activities like *taking a one-mile walk without rest* or *walk on a slippery surface*. A similar result was previously found in the physical function subscale of the EORTC QLC-30, a widely-used health-related quality of life instrument (67). In that study, participants from six East Asian countries (South Korea, Singapore, Taiwan, China, Myanmar, and Hong Kong), most of whom responded to the EORTC QLC-30 in Chinese, tended to score relatively high on two items regarding their ability to *take a short walk* and *needing to stay in bed* compared to respondents from the UK (all of whom responded in English). Per the authors, differential item functioning was primarily cultural rather than linguistic, which they concluded from their observation that Singaporeans, whom were bilingual and could choose either the English or Chinese translation, had response patterns from the English version that appeared closer to those of the East Asian countries than to English speaking countries.

It has been argued that there are more negative views on aging in China compared to the US in several life domains, including physical and mental fitness (68, 69). These cross-country differences do not appear to be solely explained by biological changes related to aging [e.g., decreased ability to perform daily tasks as people get older (69)], so higher population aging rates in China compared to the US cannot completely account for these differences. Variations in other factors like individualism/collectivism seem to also explain these East-West differences (69). Individualism has been found to be associated with more positive views on aging (68), and it has also been found to be higher in the US compared to China (70). Mandarin speakers in our sample were older compared to other languages (85.42% were 75+ years old), but Cantonese speakers had age profiles similar to English and Spanish speakers, supporting the idea that age group differences might not completely explain the observed differences in reports of difficulties performing physical activities. In contrast, all Mandarin and Cantonese speakers were foreign born, making them more likely to have cultural values associated with higher collectivism and lower individualism, which can in turn make them more likely to have negative views on aging in relation to their functioning.

We also found that the item *learn new task* was more related to the WHODAS 2.0 cognition domain among Mandarin and Cantonese speakers. The possibility of some degree of culturally determined differential functioning in this item has been previously found among rural Chinese older populations in the preceding version of the WHODAS 2.0 (the WHODAS II; 26]. Spanish speakers in the present study also seemed to report less difficulties with *day-to-day school/work* for reasons unrelated to their level of functioning. In contrast, the study by Sousa et al. (26) using the WHODAS II found no cultural differences between Spanish and Chinese speaking countries for the item *everyday activities* (which was replaced by *day-to-day school/work* in the WHODAS 2.0), suggesting that more research within Latinos and Spanish speakers in relation to this item might be needed (26).

### Conclusion and Limitations

Screening measures of anxiety, depression and level of functioning were found to be conceptualized similarly in a randomized trial sample of US minority older adults who were assessed in English, Spanish, Mandarin or Cantonese. However, at the item-level symptom burden, we identified symptoms with some degree of differential item functioning. Although our results were consistent with prior literature comparing expression of psychological symptoms across language and racial/ethnic groups (suggesting that the source of differential item functioning might be primarily cultural rather than linguistic), we singled out a non-negligible number of non-invariant items that may require careful examination before considering their use to compare symptoms of psychopathology among linguistically diverse older adult populations.

Our study has several limitations. Like prior studies using racial/ethnic diverse samples from randomized trials, we were constrained by small sample size in each language. A 2016 analytical review found that sample size and number of groups seem to be unrelated to the level of invariance achieved (11); however, that does not mean that our study could have not benefited from both an overall larger sample and a larger sample in each language group. Further, respondents in our sample all had mild to severe depression and anxiety symptoms and some degree of mobility limitations, so results may not generalize to older adults who have no psychological diagnoses and are functionally intact. We tested invariance comparing linguistic groups which, though most likely was equivalent to racial/ethnic group for Spanish, Mandarin and Cantonese speakers, did not apply to English speakers whom included both White and Black older adults. Finally, although previous studies have documented differences in the expression of psychopathology between males and females, we did not examine whether there were differences in our results by gender since testing measurement invariance across both gender and language groups was not the aim of our study. In addition, we believe that we would have not had adequate power to test for differences in our results by gender given the low number of males in each language group, particularly among English (*N* = 7) and Mandarin speakers (*N* = 9).

Despite these limitations, our study expands invariance testing in self-reported health outcome measures within psychological research. Health disparities are often measured using data from self-reported measures (17). Thus, our findings emphasize the importance of performing invariance tests before claiming that racial/ethnic differences in health outcomes exist or do not exist. In particular, the results from the present study indicate that to objectively compare levels of psychopathology between linguistically diverse older adult populations, several symptoms with some degree of differential item functioning might need to be excluded. Our findings also highlight the need for additional cross-validation studies using larger samples of different racial/ethnic and language groups, which would allow more in-depth analyses of the type of differential item functioning and the potential risk of response bias among ethnically and linguistically diverse patients.

## Data Availability Statement

The raw data supporting the conclusions of this article will be made available by the authors, without undue reservation.

## Ethics Statement

The studies involving human participants were reviewed and approved by The Institutional Review Boards of Massachusetts General Hospital/Partners HealthCare and New York University. The patients/participants provided their written informed consent to participate in this study.

## Author Contributions

MA, MC-G, and KA contributed to the conception and design of the study. MC-G organized the database and performed the statistical analysis. MC-G and MA wrote the first draft of the manuscript. IH wrote sections of the manuscript. PS advised on statistical methods. All authors contributed to manuscript revision and approved the submitted version.

## Conflict of Interest

The authors declare that the research was conducted in the absence of any commercial or financial relationships that could be construed as a potential conflict of interest.
